# Crosslinker-Based Regulation of Swelling Behavior of Poly(*N*-isopropylacrylamide) Gels in a Post-Polymerization Crosslinking System

**DOI:** 10.3390/gels6010002

**Published:** 2019-12-21

**Authors:** Shohei Ida, Akimitsu Katsurada, Mitsuhiro Tsujio, Motoharu Nakamura, Yoshitsugu Hirokawa

**Affiliations:** Department of Materials Science, The University of Shiga Prefecture, 2500 Hassaka, Hikone, Shiga 522-8533, Japan

**Keywords:** gel, thermoresponsive property, volume phase transition, post-polymerization crosslinking, chain modification, crosslinker, acrylamide derivative, swelling behavior

## Abstract

A fundamental understanding of the effect of a crosslinker on gel properties is important for the design of novel soft materials because a crosslinking is a key component of polymer gels. We focused on post-polymerization crosslinking (PPC) system utilizing activated ester chemistry, which is a powerful tool due to structural diversity of diamine crosslinkers and less susceptibility to solvent effect compared to conventional divinyl crosslinking system, to systematically evaluate the crosslinker effect on the gel properties. A variety of alkyldiamine crosslinkers was employed for the synthesis of poly(*N*-isopropylacrylamide) (PNIPAAm) gels and it was clarified that the length of alkyl chains of diamine crosslinkers strongly affected the gelation reaction and the swelling behavior. The longer crosslinker induced faster gelation and decreased the swelling degree and the response temperature in water, while the crosslinking density did not significantly change. In addition, we were able to modify the polymer chains in parallel with crosslinking by using a monoamine modifier along with a diamine crosslinker. This simultaneous chain modification during crosslinking (SMC) was demonstrated to be useful for the regulation of the crosslinking density and the swelling behavior of PNIPAAm gels.

## 1. Introduction

Crosslinking is a key reaction for construction of network structure of a polymer gel, which is a soft and wet material anticipated for various applications such as biomedical devices, actuating systems and catalyst platforms [1,2,3,4,5,6,7,8]. Properties and functions of a polymer gel are generally controlled by choosing an appropriate monomer consisting the network chains for the desired characteristics because it is a main component of polymer gels. On the other hand, a crosslinker is also important for the design of a gel structure and properties, and appropriate design of crosslinker structure can impart a unique property to a gel, although the amount of a crosslinker is relatively small compared to a monomer. For example, a cleavable crosslinker affords a stimuli-responsive degradability [9,10,11], and a crosslinking point with dynamic covalent bond can undergo structure reorganization by stimulus [12,13]. In addition, a polymeric crosslinker gives an amphiphilic network structure having unique swelling property in both of water and organic solvents [14,15,16,17,18,19]. Despite of these examples, the fundamental research on the effect of a crosslinker on gel properties is still limited particularly in the general synthetic method of vinyl polymer gels probably due to the poor structural diversity of a crosslinker, a divinyl compound. Free radical polymerization of a monomer in the presence of a crosslinker is a simple and important method for the preparation of functional gels but, when focusing on a crosslinker, the design of a divinyl compound often requires a bothersome synthetic procedure. In addition, divinyl crosslinking reaction is susceptible to the reaction solvents and produce the different network structure depending on the solvent even if using a same monomer/crosslinker combination [20]. This strong solvent effect on the reaction leads to the difficulty in the understanding the effect of a crosslinkers on gel properties even at simply changing the alkyl chain length of a crosslinker, because the solubility of a crosslinker also affects the reaction particularly in the case of a hydrogel prepared in water. As a result, the influence of the crosslinking agent on the gel has been difficult to be investigated so far in spite of the importance in gel properties [21].

**Scheme 1:**
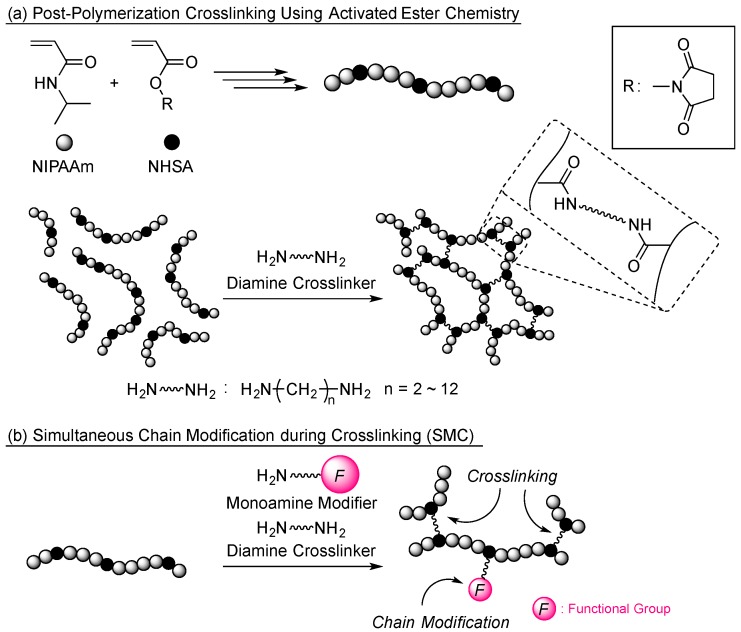
(**a**) Post-polymerization crosslinking (PPC) utilizing activated ester chemistry with a diamine crosslinker for PNIPAAm gel synthesis, and (**b**) simultaneous chain modification during crosslinking (SMC) using a functional monoamine compound as a modifier in combination with a diamine crosslinker.

We are focusing on a gel synthesis by post-polymerization crosslinking (PPC) system in which prepolymers having activated ester moieties are reacted with a diamine compound as a crosslinker (Scheme 1a) [22,23,24,25,26]. This method is performed by simply mixing a prepolymer with a crosslinker, and it is possible to diversely change the gel structure by an appropriate design of the prepolymer. For example, simple mixing two kinds of poly(acrylamide derivative) prepolymers with different solubility afforded an amphiphilic co-network structure showing unique thermoresponsive swelling behavior [24]. In addition, a designed amphiphilic structure with crosslinked domains can be obtained by using the triblock prepolymer having reactive sites in the outer blocks, and these gels turned out to exhibit large and sharp volume changes and unique mechanical properties [25,26].

Since this system uses only a simple diamine compound as a crosslinker, wider range of crosslinkers can be utilized in commercially available materials, compared to divinyl crosslinking system. In addition, a recent study on the effect of reaction condition on poly(*N*-isopropylacrylamide) (PNIPAAm) gel synthesis clarified that PPC gels are less susceptible to reaction solvents than gels prepared by general divinyl-crosslinking [23]. These facts indicate that PPC system is more suitable for fundamental understanding of the effect of crosslinker structure on gel properties rather than divinyl-crosslinking, in which the solvent effect strongly exerts and the solubility of crosslinkers is problematic particularly in the synthesis of hydrogels.

Based on these backgrounds, this study focuses on the influence of crosslinker structure on PNIPAAm gel, and a variety of diamine compounds was employed for PNIPAAm sysnthesis by PPC using activated ester chemistry. We employed an identical prepolymer and only changed the crosslinker structure to clarify the effect of a crosslinker, which is used in a small portion at gel synthesis but indispensable component for a gel. First, a variety of diamine compounds with different alkyl spacer length was examined to investigate the effect on gelation behavior and thermoresponsive swelling property in water. It revealed that the spacer length strongly affected the reaction rate of gelation and swelling behavior such as swelling degree and response temperature of PNIPAAm gels in water. In addition, a “simultaneous chain modification during crosslinking (SMC)” is able to be conducted in this PPC system by using a monoamine compounds in combination with a diamine crosslinker. In this SMC reaction, a monoamine compound can function as a chain modifier by converting activated ester units into functional groups together with crosslinking reaction by diamine (Scheme 1b). This SMC is considered to be a useful and convenient system for fine-tuning the properties of gels prepared from one series of prepolymers. Thus, we demonstrated the control of crosslinking density and swelling property of PNIPAAm gels by SMC using various monoamines with different affinity to water.

## 2. Results and Discussion

### 2.1. Prepolymer Synthesis

**Figure 1:**
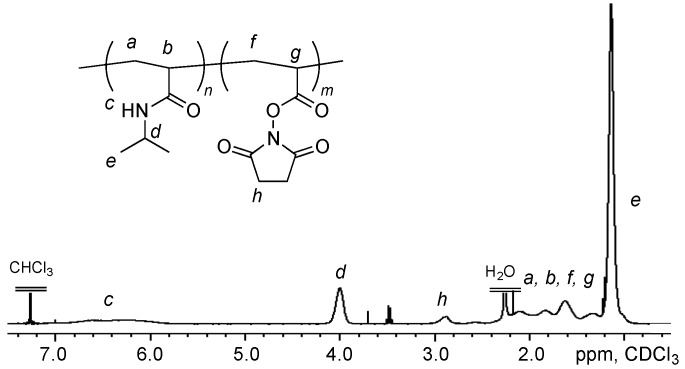
^1^H NMR spectrum of **P_N6_**.

PNIPAAm prepolymers were prepared by free radical copolymerization of NIPAAm and a small amount of the monomer carrying activated ester, *N*-(acryloyloxy)succinimide (NHSA), with azobisisobutyronitrile (AIBN) as a radical initiator in 1,4-dioxane as previously reported [23]. In this study, we prepared three kinds of prepolymers with different NHSA content as summarized in Table 1. For example, in the concentration condition of [NIPAAm]:[NHSA] = 1930:70 (mM), monomer conversion reached 52% for NIPAAm and 91% for NHSA in 1 h. Since NHSA was consumed faster than NIPAAm, the polymerization was quenched before the full consumption of NIPAAm to prevent the formation of NIPAAm homopolymer, which could not contribute to the network structure at gelation process. The obtained polymer was analyzed by ^1^H NMR (Figure 1). The NHSA content per a molecule was calculated from the peak intensity ratio of methyne proton of NIPAAm (4.0 ppm: *d*) and methylene protons of NHSA (2.9 ppm: *h*) monomeric unit, and the value was 5.8%. Thus, we denote this polymer as **P_N6_** from the content of NHSA unit. In addition, we changed the feed ratio of the monomers and obtained the polymer with fewer NHSA content (**P_N2_**; 1.8% of NHSA content) and much larger content (**P_N29_**; 29% of NHSA content).

### 2.2. Effect of Crosslinker Structure on Gelation Behavior by PPC

First, we evaluated the effect of the structure of a diamine crosslinker on PPC gel synthesis. We employed **P_N6_** as the prepolymer for the crosslinking reaction with alkyldiamines with a variety of methylene units [H_2_N-(CH_2_)_n_-NH_2_; *n* = 2–12: we denote these compounds as “nDA”] as the crosslinker in tetrahydrofuran (THF). In this gelation reaction, amino groups of the crosslinkers react with NHSA units in the prepolymer to produce amide bonds as the crosslinking points (Scheme 1a), and therefore we set the equimolar condition between amino groups in the crosslinker and NHSA units in the prepolymer. The reactions were performed under two concentration conditions: [NIPAAm unit] = 500 and 700 mM.

**Figure 2:**
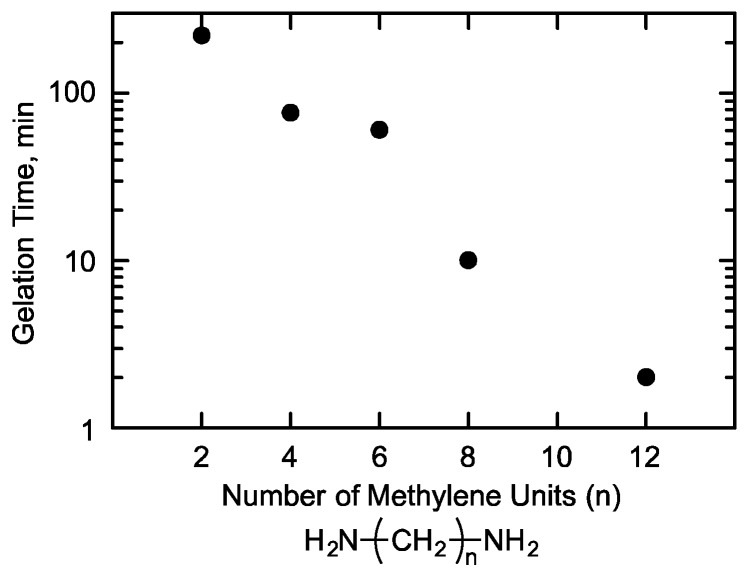
Effect of crosslinker structure on gelation time of PPC gel synthesis. Gelation condition: [NIPAAm unit in **P_N6_**] = 500 mM, [H_2_N-(CH_2_)_n_-NH_2_] = 15 mM in THF at room temperature.

All the examined crosslinkers induced gelation after the injection of crosslinkers to the prepolymer solution even in diluted condition ([NIPAAm unit] = 500 mM). The gelation time was strongly dependent on the crosslinker structure as shown in Figure 2. Longer crosslinkers gelled the whole system more quickly: 12DA with 12 methylene units (1,12-dodecanediamine) gave a gel within 2 min while gelation by 2DA (ethylenediamine) took more than 3 h. It was presumably due to the suppression of the steric hindrance of polymer chains when using a longer crosslinker. A longer crosslinker is more movable than a shorter crosslinker after one side of amine groups reacts with a prepolymer. It leads to an expansion of the distance between two prepolymers participating the crosslinking reaction, and diminishes the steric hindrance between two polymers, resulting a shorter gelation time.

**Figure 3:**
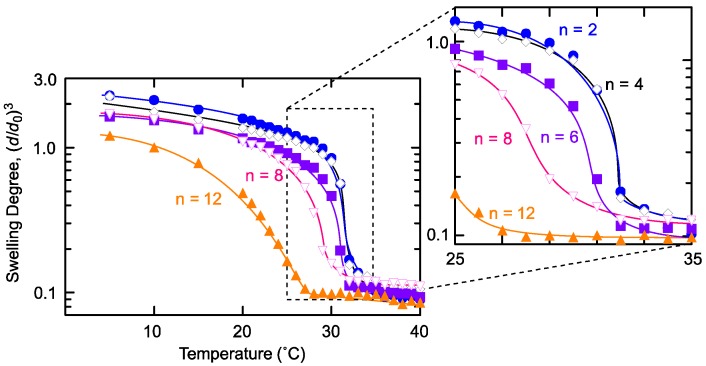
Temperature dependence of the swelling degree in water of PNIPAAm gels synthesized by PPC with diamine crosslinker with a variety of methylene units. Gelation condition: [NIPAAm unit in **P_N6_**] = 700 mM, [H_2_N-(CH_2_)_n_-NH_2_] = 22 mM in THF at room temperature.

Then, we evaluated the effect of diamine crosslinkers on the swelling behavior of PNIPAAm gels in water. The gels were prepared in THF with the concentration condition of [NIPAAm unit] = 700 mM. After the gelation, the internal solvent of the gels was replaced into water, and the temperature dependence of the swelling degree was measured (Figure 3). All the obtained PNIPAAm gels showed temperature-responsive behavior: the gels swelled at low temperature and shrunk with increasing temperature. Clear difference derived from the crosslinker structure was observed in the swelling degree at the swelling state (e.g., at 5 °C) and the shrinking temperature. The longer crosslinker gave smaller swelling degree at the swelling state, and this phenomenon could be interpreted from two factors. One is the higher crosslinking efficiency with the longer crosslinker, which corresponded to the relation with the gelation time as shown in Figure 2. The other is the hydrophobicity of the crosslinker. The longer alkyl chain of the crosslinker rendered the gel more hydrophobic, resulting in the lower swelling degree as well as lower response temperature.

**Figure 4:**
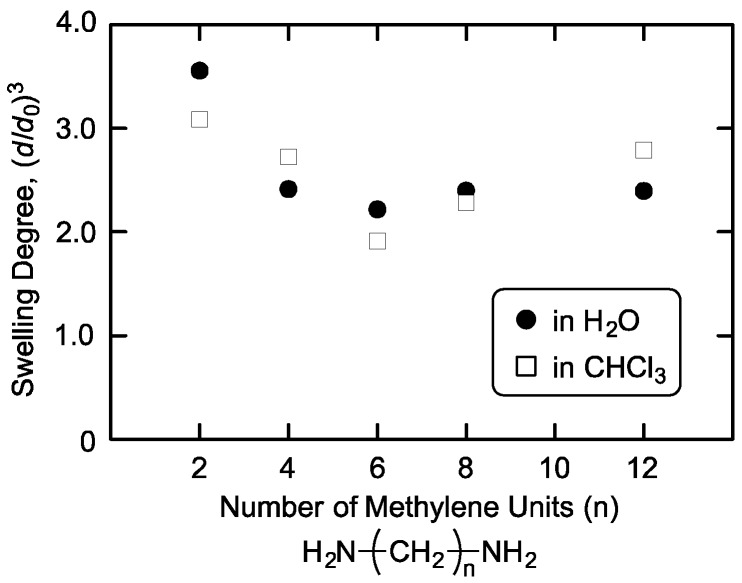
The swelling degree in water and CHCl_3_ of PNIPAAm gels synthesized by PPC with diamine crosslinker with a variety of methylene units. Gelation condition: [NIPAAm unit in **P_N2_**] = 700 mM, [H_2_N-(CH_2_)_n_-NH_2_] = 6.4 mM in THF at room temperature.

For the further evaluation of the crosslinking efficiency, swelling measurement was performed not only in water but also in an organic solvent, which is a good solvent for aliphatic crosslinking sites, using the polymer with lower NHSA content (**P_N2_**) (Figure 4). In water, only the gel prepared with 2DA showed a large swelling degree and no significant difference was observed in the other gels prepared with 4DA~12DA. Considering that the amount of the crosslinker was smaller than the case of the gels prepared from **P_N6_** shown in Figure 2, the value of the swelling degree indicates almost same crosslinking density in the case of n ≥ 4 with **P_N2_**. Then, the swelling degree in an organic solvent was examined. Among the common organic solvents, CHCl_3_ was reported to give a relatively high swelling degree of PNIPAAm gels due to high affinity to polymer chains [27]. CHCl_3_ is also considered to be a good solvent for alkyldiamine crosslinker due to low polarity and, therefore, we used it as a reference solvent for evaluation of crosslinking density of PNIPAAm gels. As a result, the similar tendency of the swelling degree was observed in CHCl_3_ with that in water. It also supports the consideration that there was no significant difference in the crosslinking density in the gels prepared with 4DA~12DA. In addition, these results suggested that the difference in the swelling degree in the gels from **P_N6_** was dominantly derived from the hydrophobic aggregation effect of the crosslinking structure.

### 2.3. Control of Crosslinking Density by SMC Method

In PPC gel synthesis utilizing activated ester chemistry, a chain modification reaction can be simultaneously conducted with a crosslinking reaction by simply adding monoamine compounds as modifiers in combination with diamine crosslinkers (Scheme 1b). This SMC reaction can be regarded as a useful method for fine-tuning of a variety of the properties of a gel prepared from a single prepolymer. Here, we employed a PNIPAAm prepolymer with high NHSA content (**P_N29_** in Table 1; NHSA content: 29%) for this SMC reaction.

**Figure 5:**
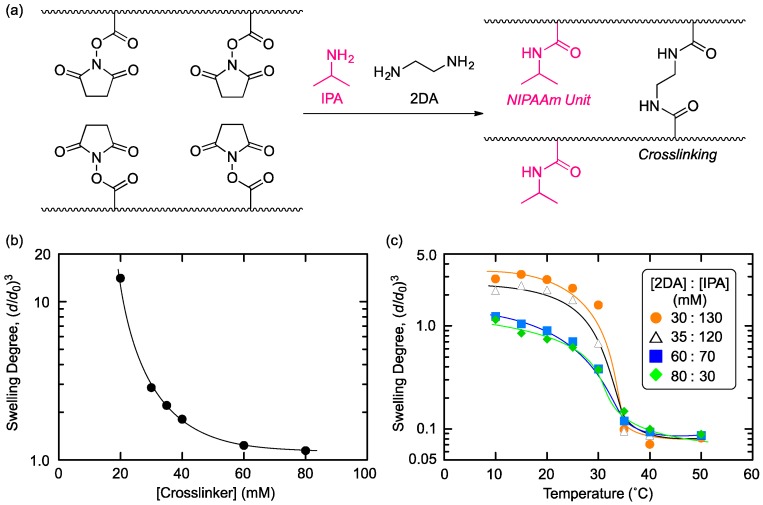
Synthesis and swelling behavior in water of PNIPAAm gels by SMC reaction in the combination of 2DA and IPA: (**a**) reaction scheme, (**b**) swelling degree at 10 °C, and (**c**) temperature dependence of swelling degree. Gelation condition: [NIPAAm unit in **P_N29_**] = 510 mM, [NHSA unit in **P_N29_**] = 190 mM, 2 × [2DA] + [IPA] = [NHSA unit] in THF at room temperature.

First, regulation of the crosslinking density of PNIPAAm gel was examined using isopropylamine (IPA) as a control modifier in combination with 2DA as a crosslinker. IPA transforms NHSA units into NIPAAm units, and the feed ratio of IPA and 2DA affects the crosslinking density with maintaining original properties of PNIPAAm gel (Figure 5a). We changed the feed ratio of IPA and 2DA under the same concentration of the prepolymer ([NIPAAm unit] = 510 mM, [NHSA unit] = 190 mM) and equimolar condition between NHSA units and amine groups. All conditions examined in this study ([2DA] = 20–80 mM) induced gelation. Among them, the lowest feed of 2DA (20 mM) gave a very soft gel with high swelling degree (>10) in cold water at 10 °C. The swelling degree at 10 °C clearly decreased with increasing the feed ratio of 2DA crosslinker (Figure 5b). These values were reflected by the crosslinking density of PNIPAAm gel because water is a good solvent for PNIPAAm chain at this temperature. Importantly, the gels showed thermoresponsive shrinking by heating at almost the same temperature (Figure 5c), indicating that polymer chains in the gels had similar characteristics regardless of the feed ratio of IPA and 2DA due to a successful transformation of NHSA units into NIPAAm units by IPA modifier. Thus, the SMC reaction with the combination of IPA and crosslinker was able to control the crosslinking density maintaining the original properties of PNIPAAm gel.

### 2.4. Control of Response Temperature by SMC Method

Then, we examined the control of thermoresponsive behavior of PNIPAAm gels by SMC reactions using monoamine compounds modifying the characteristics of the network chains. Here, hydrophobic *n*-butylamine (BA) and hydrophilic 2-hydroxyethylamine (HEA) were employed as modifiers, and the prepolymers were reacted with these modifiers in combination with 2DA crosslinker and a control modifier, IPA, transforming NHSA units into NIPAAm units ([NIPAAm unit] = 510 mM, [NHSA] = 190 mM, and [2DA] = 35 mM). The feed ratio of modifier amine (BA or HEA) and IPA was systematically changed to regulate the properties of the network chains.

**Figure 6:**
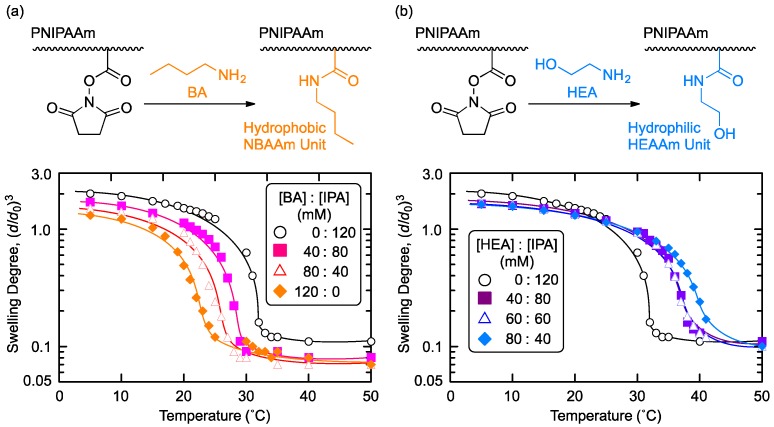
Temperature dependence of PNIPAAm gels synthesized by SMC reaction with modifier monoamines: (**a**) hydrophobic BA, and (**b**) hydrophilic HEA. Gelation condition: [NIPAAm unit in **P_N29_**] = 510 mM, [NHSA unit in **P_N29_**] = 190 mM, [2DA] = 35 mM, [modifier amine (BA or HEA)] = 120 mM in THF at room temperature.

When BA was used as a modifier, the swelling degree at low temperature such as 5 °C decreased with increasing the feed ratio of BA (Figure 6a). At the same time, the shrinking temperature clearly shifted lower (e.g., around 25 °C at [BA] = 120 mM) from 32 °C of a pristine PNIPAAm gel. In this case, NHSA units in the polymer chains transformed into hydrophobic *N*-*n*-butylacrylamide (NBAAm) units. These transformed hydrophobic units gave a less affinity to water to lead lower swelling degree and shrinking temperature. On the other hand, a modification with HEA gave higher response temperature (Figure 6b). HEA transformed NHSA units into hydrophilic hydroxyethyl acrylamide (HEAAm) units, and these hydrophilic units pushed up the shrinking temperature. Thus, we demonstrated that the chain modification and the regulation of the swelling behavior of the gels with concurrent crosslinking were able to be performed by the combination of monoamine modifier and diamine crosslinker.

## 3. Conclusions

We focused on the PPC utilizing activated ester chemistry to understand the effect of the crosslinker structure on the swelling properties of PNIPAAm gels. A variety of alkyldiamine crosslinkers was examined for the gel synthesis and it clarified that the length of alkyl spacer in the crosslinkers strongly affected the gelation time and the hydrophobicity of the network, decreasing the swelling degree and the response temperature in water. On the other hand, the crosslinking density was almost the same in the case of the gels prepared with the crosslinkers with n > 4, judged by the swelling degree in water as well as chloroform, the good solvent both for PNIPAAm and the crosslinkers. In addition, SMC reaction was examined utilizing monoamine modifier along with diamine crosslinkers. We demonstrated the regulation of the crosslinking density and the swelling behavior using appropriate modifiers. Thus, the PPC is a powerful tool not only for the fundamental understanding of the crosslinker effect on gel properties but also for the functionalization of polymer gels. This reaction system would provide the new insight for the design of novel soft materials.

## 4. Materials and Methods

### 4.1. Materials

NHSA was synthesized according to the literature [28]. NIPAAm (Wako; >98%) was recrystallized from toluene/*n*-hexane. AIBN (Wako, Osaka, Japan; >98%), 1,2,3,4-tetrahydronaphthalene (tetralin; Aldrich, St. Louis, Missouri, USA; 99%), ethylenediamine (2DA; Wako; >99%), 1,4-butanediamine (4DA; Wako; >98%), 1,6-hexanediamine (6DA; Wako; >98%), 1,8-octanediamine (8DA; Wako; >98%), 1,12-dodecanediamine (12DA; Tokyo Kasei, Tokyo, Japan; >98%), IPA (Wako; >99%), BA (Wako; >98%), HEA (Tokyo Kasei; >99%), 1,4-dioxane (Wako; >99.5%), THF (Wako; >99.5%), and DMF (Wako; >99.5%) were used as received.

### 4.2. Prepolymer Synthesis

The polymerization solution was prepared by mixing NIPAAm (4.36 g; 0.0385 mol), NHSA (236 mg; 1.40 × 10^−3^ mol), tetralin (2.0 mL), and AIBN (66 mg; 4.0 × 10^−4^ mol) in 1,4-dioxane (18.0 mL). The solution was bubbled with nitrogen for 10 min, and then heated to 60 °C in an oil bath to initiate the polymerization. After 1 h, the reaction was quenched by cooling the reaction mixture down to −78 °C. The conversion of NIPAAm and NHSA was determined by ^1^H NMR analysis from the peak intensity ratio of the peaks assignable to the residual monomers and an internal standard, tetralin. Then, the purified PNIPAAm prepolymers were obtained by reprecipitation in diethyl ether.

### 4.3. PPC-Gel Synthesis

The gelation reaction was performed in a test tube containing glass capillaries (internal diameter: 1300 μm; volume: 40 μL) to prepare the cylindrical gels with small diameter. First, NIPAAm/NHSA copolymer, **P_N6_** [NHSA content: 5.8 mol% (calculated by ^1^H NMR)] (87 mg; 7.0 × 10^−4^ mol of NIPAAm unit and 4.3 × 10^−5^ mol of NHSA unit), was dissolved in THF (0.90 mL). Then, a solution of 2DA (4.3 × 10^−5^ mol of amino groups in 0.10 mL THF) was added, and the mixture was kept at room temperature for 24 h. The gelation time was determined by tilting method checking a loss of fluidity of the solution. After the completion of the reaction, the cylindrical gels were taken out from the capillaries and washed by immersion in large amount of distilled water for several days to remove the residual molecules.

### 4.4. SMC Reaction

The gelation reaction was performed in a test tube containing glass capillaries (internal diameter: 1300 μm; volume: 40 μL) to prepare the cylindrical gels with small diameter. First, NIPAAm/NHSA copolymer, **P_N29_** [NHSA content: 29 mol% (calculated by ^1^H NMR)] (90 mg; 4.9 × 10^−4^ mol of NIPAAm unit and 2.0 × 10^−4^ mol of NHSA unit), was dissolved in DMF (0.90 mL). Then, 0.10 mL of a mixed solution of 2DA (1.2 × 10^−4^ mol of amino groups) and IPA (8.0 × 10^−5^ mol of amino groups) in DMF was added, and the mixture was kept at room temperature for 24 h. Then, the cylindrical gels were taken out from the capillaries and washed by means of immersion in large amount of distilled water for several days to remove the residual molecules.

### 4.5. Measurements

The number-averaged molecular weight (*M*_n_) and polydispersity index (*M*_w_/*M*_n_) of PNIPAAms were determined by size-exclusion chromatography (SEC) with poly(methyl methacrylate) calibration (standard samples were purchased from Agilent, Santa Clara, California, USA). DMF containing 10 mM LiBr was used as an eluent, and the measurement was conducted at 40 °C using three polystyrene gel columns (PLgel 5 μm MIXED-C, PLgel 3 μm MIXED-E and Shodex KF-805L) connected to a Shimadzu LC-10AD precision pump, a Shimadzu RID-10A refractive index detector, and a Shimadzu SPD-10A UV–VIS detector set at 250 nm (Shimadzu, Kyoto, Japan). ^1^H NMR analysis was performed with a JEOL JNM-LA400 spectrometer (JEOL, Akishima, Japan), operating at 399.65 MHz. The swelling degree of PNIPAAm gels in water was determined from the diameter of cylindrical gels using a digital microscope (MOTICAM2000, Shimadzu, Kyoto, Japan). The swelling degree was calculated by (*d*/*d*_0_)^3^; *d*_0_ is the inner diameter of the capillary and *d* is the equilibrium diameter at predetermined temperature.

## Figures and Tables

**Table 1:** Synthesis of NIPAAm/NHSA prepolymer ^1^.

Code	Feed Concentration [NIPAAm]:[NHSA] (mM)	Conversion [%]		NHSA Content [%] ^2^	*M*_n_^3^	*M*_w_/*M*_n_^3^
		NIPAAm	NHSA			
**P_N2_**	1980:20	37	58	1.8	91,000	1.51
**P_N6_**	1970:30	52	91	5.8	85,000	1.58
**P_N29_**	1500:500	73	96	29	51,000	1.93

^1^ All polymerizations were conducted with AIBN (20 mM) as initiator at 60 °C in 1,4-dioxane for 1 h.^2^ Measured by ^1^H NMR. ^3^ Measured by SEC with PMMA standard.
